# Including Research Subjects without Decision‐Making Capacity Only as Last Resort

**DOI:** 10.1002/eahr.70013

**Published:** 2026-07-02

**Authors:** Linus Broström, Anna Nilsson

**Affiliations:** ^1^ Docent (associate professor) at the section of medical ethics in the Department of Clinical Sciences, Lund, at Lund University; ^2^ Docent (associate professor) in human rights in the Department of Law in the Faculty of Law at Lund University in Sweden

**Keywords:** human research protections, capacity to consent, disability, discrimination

## Abstract

The aim of this paper is to discuss the ethical and legal justification of the broadly accepted ethical principle that researchers may not include adults *without* decision‐making capacity to consent to participating in research studies if there are potential research participants *with* capacity who could be included instead. From the perspective of disability rights, the paper argues that this ethical principle is overprotective in its current form as it gives insufficient consideration to providing persons with disabilities the opportunity to participate in research on an equal basis with others. Nonetheless, the paper also argues that rather than rejecting this ethical principle entirely, it should be qualified to strike a better balance between protecting people without decisional capacity to consent to research and including them.

Consider a study on the efficacy of a new vaccine against a seasonal virus. The vaccine is designed for the general population, but researchers are particularly interested in its effects on elderly individuals in nursing homes, as they are at a higher risk of severe illness. However, many residents in these facilities have cognitive impairments, such as dementia, and thus lack decisional capacity to provide informed consent to participate in research. The research ethics committee (REC) applies the ethical principle regarding research with humans that persons who lack capacity to consent can only be included if the research cannot be conducted with persons who do have capacity. Since there are plenty of physically vulnerable older adults in the community who *can* consent, the ethics committee insists that the study be conducted only with this group.

Consider a different study on decision‐making and risk‐taking behavior. Researchers are interested in how people of different ages and backgrounds make choices in uncertain situations, such as financial decisions, social interactions, or everyday problem‐solving. The study involves presenting participants with various hypothetical scenarios and tracking their responses to identify patterns in reasoning and risk assessment. A group of young adults with Down syndrome who have limited capacity to understand the purpose of the research and the study's potential risks express an interest in participating. However, the REC applies the ethical principle that individuals who lack capacity to provide informed consent can only be included if the research cannot be conducted with those who do have the capacity. Since the study is not specifically about Down syndrome or intellectual disabilities, and plenty of participants without cognitive impairments who have the capacity to provide informed consent are available, the committee decides that the individuals with Down syndrome who do not have decisional capacity must be excluded from the study.

The aim of this paper is to discuss the ethical and legal justification for the principle that researchers may not include adults without decisional capacity to provide informed consent to participate in research studies if there are potential research participants *with* capacity to do so. In this introductory section, we provide some brief background, and then turn to describing the relevant ethical principle, the idea behind it, and the various ways in which it is articulated in research ethics governance. We explain why current versions of this principle could be considered ethically and legally problematic and discuss the prospects of revising the principle.

First, some background. As is well known, human subjects research is extensively regulated, especially research involving non‐negligible risks and burdens to those enrolled. International treaties and regulations, domestic legislation and guidelines of professional organizations, and institutions of higher education or funding bodies, are, to a large extent, designed to offer individuals participating in research various kinds of protection. It is common knowledge that the requirement of obtaining free and informed consent from those whom researchers wish to include in their studies is a key protective provision in these legal and soft law frameworks. That is, before they are enrolled, potential research participants typically need to be provided with relevant information about the study, and the individuals must give their free and informed consent to participation.

The normative role of informed consent in research ethics regulations is never elaborated in those regulations. This lack of explicit elaboration does not distinguish research ethics regulations from other regulations in which informed consent serves a critical justificatory function. In such regulations, the ethical and legal significance attributed to informed consent must be inferred from other norms and principles undergirding the regulation rather than derived directly from the regulatory text. Several different, albeit related, ideas could be in play here. First, obtaining informed consent is useful because it allows an individual to protect their own interests, which is especially important when it comes to interests that may be hard to spot or correctly appreciate by external observers. Ensuring that consent is obtained could also promote general trust in the research enterprise, and by so doing promote cooperation—which is often a prerequisite for, or at least conducive to, successful research. But most importantly, it could be argued, when there are fundamental interests at stake for the individual—concerning health, bodily integrity, psychological well‐being, or the like—there is a prima facie moral prohibition against using people for the sake of others, against using someone as a means toward others’ ends. Consent, the idea goes, can lift that prohibition, because by relying on the individual's own authorization, one will still respect the individual as a person and as an equal, thus making whatever instrumentalization involved less objectionable. And by ensuring that the individual's consent is informed, one goes a long way toward ensuring that the consent offered does not depend on some significant epistemic vulnerability of the person. Not exploiting such vulnerabilities, it could be said, is also key to showing due respect.

Sometimes obtaining informed consent seems to be impossible. One reason for this is that some potential

**That persons with disabilities may have an interest in the opportunity to participate in research, and that they ought to have equal opportunities to do so, are not merely conceivable ethical considerations.**

research participants appear to not have the capacity to understand what needs to be understood for their consent to be considered valid, if they can form a view on the matter at all. When it comes to adults, this could be due to disease or disability; for example, traumatic brain injury, dementia, or intellectual disability could seriously impair an individual's ability to understand what the research involves and to reflect upon the pros and cons of participating. From a protection perspective, the simplest solution to this problem would be to exclude persons with such impairments from research. However, this is not an appealing option; for one thing, it would obstruct important research regarding groups of individuals that might (in the future) benefit from the research. For that reason, research regulations typically permit including in research individuals without the capacity to provide informed consent as long as certain safeguards are met. These safeguards generally include the provision of a legally authorized representative of the individual concerned—someone who can protect their interests, and consent on their behalf. Often, safeguards also include provisions to the effect that either there must be the promise of some kind of personal benefit, or the burdens and risks of harm must be “minimal” (or at most involve a “minor increase” over minimal risks and burdens).^1(gln.16)^ Whatever one thinks of the particular safeguards that current guidelines and legislation have settled on, some kinds of extra protection of the cognitively vulnerable, compensating for their lack of capacity to consent, seems imperative, especially in light of how the relevant group historically has been abused in research. Infamous examples include the Willowbrook hepatitis studies and the Vipeholm dental caries experiments. (Iacono and Carling‐Jenkins provide an overview of the historical context relevant to the development of research ethics regulations governing research involving people with intellectual disabilities.)^2^

Research governance imposing special safeguards for those without capacity to consent to research participation typically contains some provision to the effect that including such persons in research (to which the regulations or guidelines are applicable) is permissible only when that research cannot instead be conducted with persons who have capacity to consent. We shall henceforth call this provision the *last resort rule*, as it permits the inclusion in research of individuals without capacity to consent only as a last resort. The last resort rule, as we shall see, is formulated in different ways in different regulations and guidelines, and some of these differences may matter for certain purposes. In the next section, we provide an overview of international research governance instruments that include the last resort rule, along with a few examples from domestic legal systems, and remark on the potential implications of the rule being formulated in slightly different ways.

## The Last Resort Rule

At the international level, the last resort rule appears in, among others, the Convention on Human Rights and Biomedicine (Oviedo Convention),^3^ the European Union Clinical Trials Regulation,^4^ the Declaration of Helsinki (DoH),^5(para.4)^ and in the Council for International Organizations and Medical Sciences (CIOMS) and World Health Organization's International Ethical Guidelines for Health‐related Research Involving Humans.^1^ The Oviedo Convention's version of the rule can be found in Article 17, part of which says that “Research on a person without the capacity to consent as stipulated in Article 5 may be undertaken only if all the following conditions are met: … iii. research of comparable effectiveness cannot be carried out on individuals capable of giving consent.”

This is also how the last resort rule is formulated in Article 15 of the Additional Protocol to the Convention on Human Rights and Biomedicine, concerning Biomedical Research^6^ and elaborated in its explanatory report.^7(para.83)^ The last resort provision in the EU Clinical Trials Regulation, in Article 31, is articulated as: “In the case of incapacitated subjects who have not given, or have not refused to give, informed consent before the onset of their incapacity, a clinical trial may be conducted only where, in addition to the conditions set out in Article 28, all of the following conditions are met: … (e) the clinical trial is essential with respect to incapacitated subjects and data of comparable validity cannot be obtained in clinical trials on persons able to give informed consent, or by other research methods …” Similarly, CIOMS at guideline 16 stipulates that studies with no potential individual benefits for participants (outweighing the risks) should first be conducted on individuals capable of giving consent, “unless the necessary data cannot be obtained without participation of persons who are incapable of giving informed consent.”

The version of the DoH from 2013 stated that individuals who are incapable of giving informed consent must not be included in a research study unless certain criteria are satisfied, one of them being that “the research cannot instead be performed with persons capable of providing informed consent.”^8(para.28)^ That version has been replaced by the 10th revision, in which the last resort rule is phrased in a somewhat different way. The new paragraph 28 does not explicitly contain a last resort rule. It does stipulate, however, that “[t]hose persons incapable of giving free and informed consent are in situations of particular vulnerability,” and therefore they “are entitled to the corresponding safeguards.” The relevant safeguards are articulated in the new paragraphs 19 and 20. Paragraph 20, specifically, states that “[r]esearchers should only include those in situations of particular vulnerability when the research cannot be carried out in a less vulnerable group or community, or when excluding them would perpetuate or exacerbate their disparities.” While the first disjunct isn't, strictly speaking, equivalent to the last resort rule of the DoH of 2013—where the requirement was to include those with capacity to consent if possible—its reformulation as a preference for including those who are less vulnerable does not necessarily signify a substantive departure. Rather, the revised rule retains the core principle of last resort, with capacity to consent remaining a key factor in determining whether someone is considered vulnerable.

The last resort rule can also be found in domestic legislation. For example, Section 31 of the UK's Mental Capacity Act 2005^9^ states that “The appropriate body may not approve a research project for the purposes of this Act unless satisfied that the following requirements will be met in relation to research carried out as part of the project on, or in relation to, a person who lacks capacity to consent to taking part in the project (‘P’).” Among these requirements is one stating that “[t]here must be reasonable grounds for believing that research of comparable effectiveness cannot be carried out if the project has to be confined to, or relate only to, persons who have capacity to consent to taking part in it.” In addition, the Swedish Ethical Review Act (Section 21)^10^ says, in our translation, that “research concerning a research person referred to in section 20 may be carried out [only] if … the research can be expected to provide knowledge that is not possible to obtain through research with consent ….” These are just two examples, of no greater importance than other countries’ regulations, but they serve to show that some countries’ legal rules include some version of the last resort rule.

The specific circumstances under which researchers may include individuals lacking the capacity to consent are, as can be seen above, expressed differently across various regulations. The Oviedo Convention states that research with such individuals may only proceed if research of “comparable effectiveness cannot be carried out,” while the EU Clinical Trials Regulation refers to “data of comparable validity cannot be obtained,” and the Swedish ethical review legislation requires that the research will provide “knowledge that is not possible to obtain” through research with consent. Whether and to what extent these formulations impose differing thresholds is not clear. The Explanatory Report to the Oviedo Convention, for example, states that “research of comparable effectiveness” implies that the research on such individuals must be “scientifically, the sole possibility,” and that “it is not sufficient that there should be no capable volunteers.”^11(para.104)^ The report then provides a few examples of such research with individuals lacking capacity, including research aimed at improving our understanding of child development or diseases that specifically affect these populations, such as infant illnesses, or psychiatric conditions like dementia in adults. Insofar as these regulations impose distinct legal thresholds to be met and/or differences in the associated evidentiary issues, they will have implications for the level of protection afforded to participants. While these are important questions, they are of less relevance to the issues at the core of this article.

Differences between formulations and certain matters of interpretation aside, the underlying ethical idea of having research ethics governance include this kind of provision seems obvious enough. Since informed consent is considered an important element of the protection of research participants, research involving those without capacity to provide consent should indeed be the last resort—at best a necessary evil, to be permitted only when the ideal of getting consent isn't attainable. Differently put, since the lack of capacity to give informed consent implies weakened protection, it is certainly better to involve those with capacity, all else being equal, which is why researchers ought to do so. We will now discuss whether the last resort rule is, all things considered, justified.

## Ethical and Legal Considerations Challenging the Last Resort Rule

### Ethical considerations

As a fairly thin principle akin to general principles of risk minimization or least restrictive means, the spirit of the last resort rule should be uncontroversial: Surely it is better to have consent than not to have consent, all else being equal. Obtaining informed consent, as we said, arguably lifts the default constraint against using people, when the stakes are high enough for them, for the sake of others. It allows individuals to protect their own interests, and serves the broader purpose of signalling to the general public that they can *trust* that certain things won't be done to them unless they authorize the enrollment or certain important public interests can only be served by researchers deviating from the relevant norm. However, all things are seldom equal. This is reflected in the different normative placement of the last resort rule. In the 2013 version of the DoH and in the CIOMS guidelines, the rule is introduced specifically in connection with research that offers no prospect of individual benefit to the participants. By contrast, in the Oviedo Convention, the Clinical Trials Regulation, and the domestic legislation referenced above, the rule is situated differently and appears to have a broader scope. In the 2013 DoH, the last resort rule was included in paragraph 28, which reads: “For a potential research subject who is incapable of giving informed consent, the physician must seek informed consent from the legally authorised representative. These individuals must not be included in a research study that has no likelihood of benefit for them unless it is intended to promote the health of the group represented by the potential subject, the research cannot instead be performed with persons capable of providing informed consent, and the research entails only minimal risk and minimal burden.”

Here, in other words, the last resort requirement applies only to research where there is “no likelihood of benefit” for the potential research participants. The reasoning behind this provision was perhaps that it would be ethically hard to justify a requirement to the effect that those with capacity to consent must always be selected when there is a significant likelihood that persons without capacity have more to gain from being included than from being excluded. Similarly, the CIOMS guidelines also introduce a version of the last resort rule only in research where there are no expected benefits for the research participant. Guideline 16 states that “For research interventions or procedures that have no potential individual benefits for participants, two conditions apply: [1] the interventions and procedures should be studied first in persons who can give consent when these interventions and procedures target conditions that affect persons who are not capable of giving informed consent as well as those who are capable, unless the necessary data cannot be obtained without participation of persons who are incapable of giving informed consent; and [2] the risks must be minimized and no more than minimal.”

The case where there might be a direct benefit associated with participation is not the only case where all else might not be equal. What could also be at stake is an individual's interest in, perhaps even right to, opportunities to be included in research. Why would individuals be disadvantaged if they do not get such opportunities? First, they could be disadvantaged if (they have reason to believe that) actual participation would be in their interest. The (perceived) possible benefits of participation may go well beyond more narrow readings of the direct benefit condition, which are typically about positive therapeutic consequences of a research intervention, and beyond any clinical benefits that might eventually come from the findings of the study. Article 17.1(ii) of the Oviedo Convention refers to real and direct benefit to the *health* of the person. Similarly, the commentary text to CIOMS Guideline 4 on individual benefits and risks in research speaks about “clinical benefits.”^1^, ^12^ For example, research participants may be remunerated, learn more about themselves and their situation, or receive some other “collateral” benefit. Another possible benefit is that persons with impaired decision‐making capacity, like others, may have an interest in contributing to society's pursuit of knowledge, just as they may have an interest in other ways of discharging their (self‐perceived) moral obligations toward others; the literature suggests that participation in at least some kinds of research is indeed sometimes based on altruistic considerations.^13^, ^14^, ^15^, ^16^ Or they may have no thoughts at all about doing good, but hope and believe that participating in the study will turn out to be fun or interesting. In the next section we will, in effect, caution against making too much of these putative benefits of participation, but in many scenarios they certainly are possible.

Second, and this is the more important point, having the same opportunities as others may be in one's interest, regardless of what benefits might be associated with the particular options offered. Individuals who do not get the same opportunities as fellow human beings quite often feel diminished and unfairly treated. While what the opportunities are opportunities for—the particular outcomes that they make possible—may or may not be desirable, feeling like an equal can generally be assumed to be of enormous importance to everybody, and when one is excluded from choices and experiences that others get to have, for reasons one does not understand or would not approve of, the feeling of being treated as someone inferior or not really belonging could cut deep. This is a core idea in equality law and philosophy, that people should be given equal opportunities to participate in various social arenas and not be excluded for reasons linked to a disability or similar, as this implies that they are inferior and not entitled to the same level of participation in society.^17^, ^18^, ^19^

Furthermore, for many people with intellectual, cognitive, or psychosocial disabilities such exclusion is common—a pattern—arguably making the harm significantly greater than a single precluded opportunity would be. These, of course, are subjective experiential harms, but self‐determination, liberty, and nondomination are candidates for being key elements of objectively good lives,^20^ too, so there are several conceivable routes to the conclusion that persons with disabilities may have an interest in the opportunity to participate in research, especially when others have this option. That is, whether or not research participation can be regarded as a benefit, arrangements that deny disabled persons opportunities that others have could, in themselves, be negative for persons with disabilities. Similar arguments have been made in relation to ethnic or racial minorities, although the focus has mainly (but not exclusively) been on the problem with health disparities due to selection bias, rather than individuals’ interest in, or plain right to, having participation opportunities.^21^

### The human rights law perspective

That persons with disabilities may have an interest in the opportunity to participate in research, and that they ought to have equal opportunities to do so, are not merely conceivable ethical considerations. To a significant degree, this is the perspective of the UN Convention of the Rights of Persons with Disabilities (CRPD)—an international treaty, to which an overwhelming majority of nations are party.^22^ Developed in the beginning of the 21st century, the CRPD reflects an emerging consensus that the existing human rights framework had failed to protect the rights of this part of the population. Though previous human rights treaties set forth a structure that could serve to promote and protect the rights of persons with disabilities, the full potential of these treaties have not been realized.^22(preamble, para.[d],[k])^ To address this gap, a new treaty was negotiated, affirming persons with disabilities as rights‐holders and specifying the obligations of states toward them. The CRPD aims to ensure that persons with disabilities can fully enjoy their human rights on an equal basis with others.

Several provisions of the CRPD safeguard persons with disabilities from unethical research and enrollment in studies against their will. Article 15, for example, confirms that the general prohibition of medical and scientific experimentation without the free consent of the person concerned applies also to individuals with disabilities. The treaty also guarantees the right to respect for personal integrity and obliges states to protect persons with disabilities from exploitation and abuse.^22(art.16-17)^ While the CRPD clearly asserts that research participants with disabilities deserve the same protection from harm and exploitation as all participants, other key values, such as respect for individual autonomy, participation, and inclusion in the community, are arguably more central to its mission.

The perhaps most contentious right granted in the CRPD is the right to *legal capacity*, in Article 12(2). Most adults are granted the formal right (as well as practical opportunities) to make decisions that are recognized by the legal system—to purchase goods and services, to enter into contracts and other formal agreements, to provide informed consent, etc. They are not only acknowledged to be subjects under the law, but *actors*—they have legal capacity. Historically, however, adults with impaired cognition have often been deprived of these possibilities, be it through some general declaration of legal “incompetence” or “incapacity,” or just by having their attempts to act with legal consequences disqualified individually. Whether out of a perceived need to protect the vulnerable, to protect third parties, or for other reasons, the actions of those with sufficiently impaired cognitive abilities have been determined not to “count” in the eyes of the law. Article 12(2) of the CRPD is generally believed to go against this view and practice. While the precise meaning and implications of this right to be considered an actor under the law on an equal basis with others continues to be debated, legally invalidating decisions because of an individual's mental impairments is therefore considered, by many, a human rights violation. The article is thus also at odds with the research governance under discussion, which assumes that some groups of adults lack the capacity to make legally valid decisions about research participation.^23^, ^24^, ^25^

More important for the purposes of the present discussion, however, is the prohibition against discrimination in Article 5. This article obliges states parties to ensure that persons with disabilities are entitled to equal protection and benefit of the law. To this end, states shall prohibit all discrimination on the basis of disability. Article 2 of the CRPD defines disability‐based discrimination as follows: “any distinction, exclusion or restriction on the basis of disability which has the purpose or effect of impairing or nullifying the recognition, enjoyment or exercise, on an equal basis with others, of all human rights and fundamental freedoms in the political, economic, social, cultural, civil or any other field.”

A literal interpretation of this definition suggests that a practice must aim at, or have, a negative impact on the enjoyment or exercise of a human right to qualify as discrimination. No human rights treaty to date includes a right to be invited to, or participate in, a particular research study. This does not mean that the exclusion of persons with disabilities from research falls outside the scope of the CRPD's protection against discrimination.^26^ Article 5 of the CRPD is modelled on Article 26 of the International Covenant on Civil and Political Rights and thus includes a freestanding prohibition on discriminatory domestic legislation and practices in any field regulated and protected by public authorities.^27^ This implies that the prohibition of discrimination encompasses a right to equal participation in the development of new knowledge; when research studies are conducted, they must be equally open and accessible to prospective participants with disabilities.

To what extent, then, are legal frameworks containing the last resort rule consistent with the CRPD? First, this rule seems to rely on a distinction (between those who are able to consent and those who are not) that may well, as we have already mentioned, conflict with Article 12(2) of the CRPD. As already indicated, Article 12's recognition of every (adult) person's right to enjoy legal capacity has generally been interpreted as disqualifying legal orders in which persons with intellectual, cognitive, or psychosocial disabilities are held to be incapable of providing valid consent. Instead, to the extent that states’ parties insist on requiring a certain level of understanding and rationality for consent to research participation to be recognized as legally valid, they must provide access to the support needed for persons with disabilities to attain the relevant thresholds. Second, and more importantly, in a range of real‐world scenarios, the last resort rule does make it more difficult for those who have been deemed unable to consent to get opportunities to be included in research. Persons with disabilities who otherwise meet the inclusion criteria of a research study will be excluded when persons with capacity to consent are available, meaning that there will be fewer participation opportunities for the former.

### Balancing the rights and interests at stake

Whether the last resort rule violates the CRPD depends on whether the form of restriction or exclusion in question can be justified. Answering this conclusively requires thorough normative analysis, in accordance with the principles for interpreting human rights law, to determine whether the last resort rule provisions serve legitimate aims, the extent to which they contribute to those aims, whether they represent the least restrictive means to achieve them, and, finally, whether they are reasonable.^28(p.37-42)^ Judgments of reasonableness ensure that the practices under review are fair and do not impose undue burdens on those affected, taking all relevant considerations into account. The UN treaty bodies have sometimes couched their reasoning in terms of “proportionality.”^28^, ^29^ This includes weighing the importance of the rights and interests at stake—on one hand, the right to legal capacity, equal participation in the development of new knowledge, and the right not to be excluded from research studies due to one's impairment or disability; and, on the other hand, the need to protect individuals with limited cognitive or intellectual capacities and to promote trust in the research process. Consideration is also given to the positive and negative effects of the last resort rule under review, including harms and wrongs like disrespect for human dignity, stereotyping and prejudice, and social exclusion.^28(p.116-122)^ Under international human rights law, there is something particularly objectionable about infringing human rights on the basis of sex, race, disability, and so on, as opposed to restricting rights for other reasons. As Khaitan puts it, “disadvantage acquires a special character when it attaches itself to groups rather than when it is distributed randomly to individuals across all groups.”^30(p.41)^ Being excluded from a research study because one does not meet the inclusion criteria is one thing; being excluded because of one's impairment or disability is normatively different—even if the factual consequence is the same. It brings about another form of harm, which needs to be taken into account in balancing the rights and interests at stake.

The regulations and guidelines involving a last resort rule mainly govern research within medicine, biomedicine, and health care, which involve studies of very different kinds, associated with very different burdens and levels of risk. No doubt, such research can pose a real danger to those participating in it. First‐in‐human drug trials, experimental neurosurgery, or research on the effects of exposure to dangerous pathogens are familiar examples of such high‐risk research. And even if they do not necessarily entail significant risks of harm, quite a few studies could still be very burdensome to those who decide to participate. Research participation requiring long‐term hospitalization; adherence to strict diets; likely sleep deprivation; or repeated biopsies or drawing of blood, could be cases in point. For any research of these kinds, imposing a last resort rule seems, intuitively, called for.

At the other end of the spectrum, there is much research covered by the last resort rule in research ethics governance relevant frameworks (including the last resort rule) that is not only “noninvasive,” but very unlikely to involve significant risks, and only associated with minor discomfort, if any at all, and at most limited privacy intrusions. Many studies will only involve routine physical exams, a single simple blood sampling, collection of saliva or hair, standard monitoring data (EKG, EEG, etc.), limited standard radiological examinations, basic skills tests, moderate exercise interventions, analyses of previously collected biological material, or questionnaires and interviews about marginally sensitive topics. For these (ubiquitous) kinds of research, the outcome of a proportionality analysis—ethical or legal—would arguably be that a last resort rule isn't reasonable, since protecting persons with disabilities from the small risks, burdens, and privacy intrusions typically involved makes little sense in the light of the price that has to be paid, such as depriving persons with disabilities—a group already deprived of similar rights and opportunities—of the right to self‐determination and the possibility to participate on an equal basis with others.

To sum up, if this analysis is correct, even approximately, a few key conclusions can be drawn. First, even when persons have a significant interest in the opportunity to participate in a research study, and the risks and burdens are very small, today's rules will often require the exclusion of persons without capacity to consent. Second, excluding the relevant individuals in such low‐risk situations is, arguably, not proportionate to the little protection needed. Third, for all that is known, the number of cases in which exclusion is disproportionate to what it might safeguard, given the individual's interest in having the opportunity to be enrolled, could make it hard to justify the last resort rule as a general, unqualified rule. We now move to discussing what a responsible revision of this rule might look like.

## Possible Ways Forward

### To whom may a revised last resort rule apply?

To the extent that the last resort rule conflicts with the CRPD, nation states that are bound by the treaty have legal reasons to enact legislation that either drops or revises this provision. All nation states that have ratified human rights treaties have an obligation to take all appropriate measures to ensure that domestic law, policy, and practice fully comply with the human rights standards set out in the treaty in question.^22 (art.4.1[a],[b])^ Similarly, to the extent the last resort rule fails to give ethically appropriate consideration to the interests of (some) persons with disabilities in having an equal opportunity to participate in research, the rule ought to be dropped or revised. How, if at all, could this be done without sacrificing important protections? In the best of all worlds, a proportionality assessment could be carried out in each individual case, determining whether a potential research participant with disabilities’ interest in having the opportunity to participate in a particular study outweighs the ethical concerns associated with his or her inclusion in that study at that particular time. However, that world is utopian: it is not feasible to make such determinations reliably in individual cases, and it is therefore inappropriate for regulations to suggest that we should try to. We therefore need a more general, and inevitably cruder, rule, one which would yield some unwelcome outcomes when applied across the board, but overall would get the balance right. Such a rule, we suggest, should apply only to those for whom it is a *reasonable presumption* that they have, or could have, some interest in the opportunity to participate. Just who belongs to this group needs to be further explored, but our discussion in this section will be based on two suggested restrictions.

First, as we mentioned previously, someone might be inclined to adopt the position that a person could have an *objective* interest in having certain opportunities or liberties unrelated to whatever interest they might have in using those opportunities or in subjectively experiencing them. We propose that a revised last resort rule should not rely on such a view. It is highly contested, and it is inadvisable to introduce research ethics provisions relying on philosophical assumptions on which there is so little agreement. This is especially so when researchers inevitably have some conflict of interest in wanting to enroll participants without the capacity to consent in their study. In other words, while it certainly is conceivable that people have an objective interest in the chance to be enrolled in research, persons with disabilities should not generally be presumed to have such an interest, and a revised last resort rule should not be applied based on such a presumption. For example, a person who has a profound intellectual disability (or perhaps even more obvious, is in a persistent vegetative state) should never, as far as the scope of the last resort rule is concerned, be assumed to have an objective interest in having the “opportunity” to be included in a study, as such an assumption not only would be highly speculative, but dangerous, given the exploitation risk that comes with research.

Second, persons with disabilities should not generally be assumed to have an interest in the research participation itself, or in whatever benefits participation might bring. When we remind ourselves that research is inherently instrumentalizing, primarily being conducted not for the research participant's sake; that there are all kinds of research; and that people differ wildly in what particular enterprises and activities they value, there is little basis for a default expectation that persons with disabilities have an interest in participating. Any assumptions of a participation interest should rather be based on strong evidence in individual cases. But while the presumption that people have interest in actual research participation would be very problematic, our contention is that the presumption that people have an interest in the *equal opportunity* to participate, is less so, given people's indisputable general wish to be treated fairly and with respect.

This leaves us with the group of individuals who quite conceivably could subjectively appreciate having the opportunity to participate in research, and/or who might have an experiential interest in that opportunity. Without knowing the individual psychologies of prospective participants, how could one reliably determine whether they belong to the group of people for whom it is a reasonable presumption that they have an interest in participation opportunities? We would suggest that having the *capacity to assent* to research participation—to have the capacity say “yes” to it with a basic understanding of what one says yes to (although it falls short of the understanding that would be required for valid consent)—is a good enough indicator of having the relevant interest.^31^ Differently put, if one has the basic grasp required for one's approval to count as assent to research participation, one is likely enough to feel excluded and wronged if there is no opportunity to be enrolled in a study that is open to others. And, conversely, if a person cannot grasp such things, in most circumstances they will probably not feel discriminated against when they are excluded from a study on the basis of the last resort rule. Notice, however, that the suggestion is not that assent itself should be required or otherwise play an important role in the protection of individuals without capacity; here we express no views on whether it should. It is that the capacity for assent may serve as a decent indicator of having an interest in being offered an opportunity to be enrolled.

### Should we drop the last resort rule?

With these caveats regarding to whom revised regulations should apply, consider first the option of dropping the last resort rule altogether, but with the common (disjunctive) requirement of (“direct” clinical) benefit or minimal risks and burdens still in place. This, in other words, would permit including persons without capacity to consent even if persons with capacity could have been enrolled instead, as long as there is a sufficient potential for the relevant kind of direct benefit or risks and burdens are minimal (and other standard safeguards like informed consent from a legal representative and the person concerned does not object remain). A revised Article 17 of the Oviedo Convention would, for example, not include its current condition, iii: “Research on a person without the capacity to consent as stipulated in Article 5 may be undertaken only if all the following conditions are met: i. the conditions laid down in Article 16, sub‐paragraphs i to iv, are fulfilled; ii. the results of the research have the potential to produce real and direct benefit to his or her health; [iii. research of comparable effectiveness cannot be carried out on individuals capable of giving consent]; iv. the necessary authorisation provided for under Article 6 has been given specifically and in writing; and v. the person concerned does not object.”^3^

What would the ethical cost be? If the benefit condition would be met only when there is little doubt of a strong probability of a net benefit to the person concerned, the cost of dropping the last resort rule might be negligible when that condition is met. At most, it could be argued, there is a sense in which the research participant would still be troublingly used as a means to others’ ends, as the well‐being of the research participant would still not be the primary reason for including them in the study. Reasonable guarantees of such well‐being would be a major constraint on participant selection, though, so the instrumentalization involved would arguably be less objectionable. However, it is not always clear just how the benefit condition should be understood in various codes,^12^, ^32^ and there are indications that sometimes it should be understood as a significantly weaker principle. For example, it is not clear that in the Oviedo Convention, there being a potential for a direct benefit implies that it is more likely than not that such a benefit will materialize. And even if one were to clarify that the research intervention needs to be “at least as advantageous” as other available interventions (CIOMS), or that inclusion must be “likely” (a probability greater than 0.5) to personally benefit the participant (DoH), it could still be a virtual coin toss whether the person without capacity to consent will benefit from being enrolled. Neither can we conclude that whatever number we should put to the relevant probability, the (epistemic) certainty with which a probability assignment is made needs to be particularly strong. This means that doing away with the last resort rule would allow for including research subjects without capacity to consent in studies where the risks and burdens are (conceivably much) greater than minimal, and the likelihood of a direct benefit is either very uncertain or barely exceeding 50%, or both. That, in turns, means there would be very little protection of this group of vulnerable research subjects, and while their rights to participation in society may not have been given the weight it deserves, just dropping the last resort rule, with no other adjustments to the relevant safeguards would, depending on the particular regulation, arguably put them at an unacceptable risk of harm.

Next, one might consider going down the CIOMS (and former DoH) route and not impose the last resort rule when there is a potential for direct benefit but impose it in all other situations. This would be problematic for the same reason that dropping the last resort rule altogether would be: It would allow for including persons without capacity to consent in cases where risks and burdens are significant while benefits for the individual included are far from guaranteed. This not only allows for risky and burdensome research enrollment when the potential benefits are not sufficiently likely, but also fails to allow for research participation in situations where not much harm could be done to willing individuals.

### Set a limit on the risks and burdens involved?

As already indicated, the benefit condition could be strengthened, or clarified, to mean, for example, that there must be, roughly, very reliable evidence of a very strong probability of a net benefit. Absent this, however, if we were to drop the last resort rule with a reasonable degree of protection still in place, one way forward would be to set an absolute limit on risks and burdens that cannot be waived by reference to any promise of benefits. First, we might simply try dropping the last resort rule in cases where risks and burdens are minimal (or involve only a minor increase over minimal risks) but keep it in place in all other cases. As such, a revision modelled on the Oviedo Convention's version of the last resort rule would read: If risks and burdens are more than minimal, research on a person without the capacity to consent may be undertaken only if research of comparable effectiveness cannot be carried out on individuals capable of giving consent.

When it comes to traditional protection concerns, the ethical cost of such a revision would arguably be twofold. First, it would allow for some risks and burdens to those who presumably are less well equipped than other available research participants to protect themselves against physical, psychological, emotional, or other harms and burdens. Second, and again, it would allow for some potentially objectionable instrumentalization of persons without capacity to consent. We must therefore address whether this way of changing the last resort rule would, for these reasons, offer too little protection.

Beginning with burdens and risks of harm, if risks and burdens are truly minimal, it follows that participants won't be “seriously harmed,” in any ordinary sense of that expression. More importantly, we contend that however one might wish to characterize the relevant harms, not getting the same opportunities as others do in society out of paternalistic concerns is generally likely to cause more harm to the relevant group than the harm inflicted upon them in minimal risk research.

As to instrumentalization, if the potential research participants have sufficient understanding to be able to assent to research participation, they will be problematically used as a means toward others’ ends only to a limited extent. These persons can be regarded as agents, who accept being used in the pursuit of new knowledge, albeit with a diminished understanding of just what they are accepting. If the capacity to understand what research is and/or what the particular study involves does not reach that assent threshold, the research subjects would indeed be significantly instrumentalized. As we have suggested, in that situation we may not be able to presume a participation interest, and the last resort rule ought to apply.

Turning things around, the opposite charge could also be made: that permitting the inclusion of persons without capacity to consent only when risks and burdens are minimal is “patronizing,” or at least gives too little opportunity for participation. Beginning with the former, the worry is that allowing persons with disability to participate only when there is virtually nothing at stake is discriminatory. In the disability movement, this point is frequently made in terms of the “dignity of risk,” the idea that it is a key element of everyone's right to self‐determination that one may end up making decisions that are in various ways unwise, decisions that will frustrate one's interests rather than serve them.^33^, ^34^, ^35^, ^36^ Letting the last resort rule go only when risks and burdens are minimal, the criticism goes, does not take seriously enough this idea about the dignity of risk. The merits of this objection will depend in part on what is meant by “minimal risk” and “minimal burden.” While these words might suggest a totally negligible negative impact on the research participant, they might be somewhat deceiving. Risks and burdens considered minimal by the applicable legal frameworks could, depending on the situation and individual sensitivities, be perceived as anything but by some of the individuals concerned. Examples of minimal risks and burdens include registering someone's weight; simple blood collection; the testing of comprehension or problem‐solving skills; and being video recorded in a public space. Perhaps it is misleading to say that there is virtually nothing at stake for those who have to endure such things. Assuming that appearances can indeed be deceiving, and “minimal” risks and burdens might not be that minimal, the easiest remedy might be to describe the relevant levels of risk and burdens with other words.

Assuming instead that so‐called minimal risks and burdens are appropriately labelled as such, the charge that it would be patronizing to drop the last resort rule (only) in such cases would have to be dealt with in some other way, if one believes that the charge has some merit. The proposals discussed so far are all conservative in the sense that they try to revise the last resort rule with no other resources than the conceptual categories and provisions already in place in current research ethics governance. It may well be, however, that a more defensible revision, one that takes into account the dignity of risk, will need to involve more nuances than these categories and provisions allow. For example, we have already mentioned the possibility of invoking a strengthened notion of potential for direct benefit. Even more promising, perhaps, when revising the last resort rule, we might try to invoke some notions of *minor* or even *moderate* risks and burdens—more than minimal, but less than great. With such additional risk levels at one's disposal, one might consider imposing the last resort rule only in research where risks are more than minor or moderate. A rule revised this way might read: If risks and burdens are more than *minor/moderate*, research on a person without the capacity to consent may be undertaken only if research of comparable effectiveness cannot be carried out on individuals capable of giving consent.

How this kind of rule would play out in concrete cases will depend on the particulars of those cases. But just to illustrate, let us return to the examples with which we introduced the last resort rule at the beginning. The psychological study on risk taking, looking into participants’ responses to hypothetical scenarios, could well turn out to be a true minimal risk study. Absent any specific reason to believe that eliciting such responses from people with Down syndrome who don't have decisional capacity would expose them to any significant psychological risks, it surely would be. If that is the case, the revised last resort rule would not exclude these individuals from the study, given that they could be assumed to have an interest in being given the opportunity to participate. The vaccination study on dementia patients in nursing homes, on the other hand, could end up being a closer call, depending both on the details of this trial, and on one's views of the acceptability of risks. Again, researchers would first have to assess which of the individuals at the relevant nursing homes could have an interest in being provided with a participation opportunity. And then the critical question would be whether the risks associated with participating in a phase III trial (with respect to possible negative side effects, for instance) exceed those that would permit enrolling persons without capacity to consent according to the modified last resort rule—something that would depend on the exact nature and size of those risks, but also on where precisely the relevant risk threshold should be set.

It goes without saying that the proposed revised last resort rule could not be fully assessed until the relevant risk levels are clarified. However, if one were to reserve the rule for more risky and burdensome research, the relevant regulation would need to be more fine‐grained in other respects, too, in order to compensate for the loss of protection brought about by such a revision of the last resort rule. Specifically, the stronger the participation interest an individual has, and the more he or she understands about the research (even if not enough in relation to traditional capacity thresholds), the more acceptable such a reform would seem to be. Conversely, the weaker the participation interest and the poorer the understanding, the more worried we ought to be about relaxing the safeguards in the way suggested. It remains to be explored just how fine‐grained research ethics regulations can realistically be made, in these and related regards, and where we are better off drawing the lines in cruder ways.

The particular way of facilitating more participation opportunities considered here, involving the imposition of absolute risk thresholds in a relaxed last resort rule, may not be the only viable one. For example, one alternative rule might state that persons who are able to assent, i.e., able to express a preference to participation in a research study with a basic understanding of what such participation implies for them, but who lack capacity for valid consent, could be enrolled in research only if a sufficient amount of the participants in the relevant study—the majority, perhaps—are individuals capable of giving consent.^37^ Such a rule would serve to protect the cognitively impaired from being intentionally exploited in research that others wouldn't voluntarily participate in. It would provide for participation opportunities, while virtually shutting the door to the kinds of research abuses those with various mental disabilities have been victim in the past. Related, it could be argued that such a rule, if followed, would lessen the likelihood that the particular individuals who are enrolled without having provided proper informed consent will be harmed or excessively burdened in the research, as risky and burdensome research would not attract that many people with the capacity to provide informed consent. It is doubtful, however, that this approach would be superior. While it would evade the challenge of setting a non‐arbitrary risk threshold, it would be vulnerable to there possibly being excessive risk takers with cognitive capacity, or participants having consented but done so because of lack of acceptable alternatives (making them exploitable), justifying problematic inclusion of those who lack the requisite cognitive capacity. But considered as a *complement* to a relaxed last resort rule, it would have merits.

## Conclusion

If protection of research participants and the facilitation of valuable research were the only relevant considerations, including a last resort rule in research ethics governance could be seen as an obvious choice, and current codes and regulations would do the job. Even if not, the importance of protecting cognitively vulnerable participants strongly suggests that some version of the last resort rule should keep playing a role in the relevant regulations and guidelines. However, once one recognizes some persons’ with disabilities interest in, and right to, *inclusion* in society on an equal basis with others—and recognizes the way in which the last resort rule can sometimes be an obstacle to the realization of such interests and rights—the rule begins to look a little problematic. Designing it in a way that mitigates these concerns thus becomes important. The ideal way of doing that remains to be worked out, but we believe the considerations presented in this paper point us in the right direction.

## ACKNOWLEDGMENTS

This article was written within the research project “Non‐discriminatory research ethics. Law reform and implementation challenges in the light of the UN Convention on the Rights of Persons with Disabilities,” funded by Riksbankens Jubileumsfond (FOE20‐0013), a Swedish independent foundation with the goal of promoting and supporting research in the humanities and social sciences. We are grateful to the anonymous reviewer for constructive comments. Thanks also to the medical ethics seminar at Lund University.
